# Rivaroxaban for stroke patients with antiphospholipid syndrome (RISAPS): protocol for a randomized controlled, phase IIb proof-of-principle trial

**DOI:** 10.1016/j.rpth.2024.102468

**Published:** 2024-06-05

**Authors:** Prabal Mittal, Rafael Gafoor, Zara Sayar, Maria Efthymiou, Ibrahim Tohidi-Esfahani, Stella Appiah-Cubi, Deepa J. Arachchillage, David Atkinson, Ekaterina Bordea, M. Jorge Cardoso, Emilia Caverly, Arvind Chandratheva, Marisa Chau, Nick Freemantle, Carolyn Gates, H. Rolf Ja¨ger, Arvind Kaul, Chris Mitchell, Hanh Nguyen, Bunis Packham, Jaye Paskell, Jignesh P. Patel, Chris Round, Giovanni Sanna, Abbas Zaidi, David J. Werring, David Isenberg, Hannah Cohen

**Affiliations:** 1 Department of Haematology, University College London Hospitals NHS Foundation Trust, London, United Kingdom; 2 Department of Haematology, Cancer Institute, University College London, London, United Kingdom; 3 Comprehensive Clinical Trials Unit, University College London, London, United Kingdom; 4 Department of Haematology, Whittington Health NHS Trust, London, United Kingdom; 5 Department of Haematology, Epsom and St Heliers University Hospitals NHS Trust, Epsom, United Kingdom; 6 Centre for Haematology, Department of Immunology and Inflammation, Imperial College London, London, United Kingdom; 7 Department of Haematology, Imperial College Healthcare NHS Trust, London, United Kingdom; 8 Centre for Medical Imaging, Division of Medicine, University College London, London, United Kingdom; 9 Department of Biomedical Engineering, School of Biomedical Engineering and Imaging Sciences, King's College London, London, United Kingdom; 10 Stroke Research Centre, University College London Queen Square Institute of Neurology, London, United Kingdom; 11 Comprehensive Stroke Service, University College London Hospitals NHS Foundation Trust, London, United Kingdom; 12 Neuroradiological Academic Unit, Department of Brain Repair and Rehabilitation, University College London Institute of Neurology, London, United Kingdom; 13 Department of Rheumatology, St George's Healthcare NHS Trust, London, United Kingdom; 14 Department of Haematology, North Middlesex University Hospital NHS Trust, London, United Kingdom; 15 Thrombosis and Anticoagulation service, Royal Free London Hospital NHS Foundation Trust, London, United Kingdom; 16 Department of Haematological Medicine, King’s College Hospital and Institute of Pharmaceutical Science, King’s College London, London, United Kingdom; 17 Louise Coote Lupus Unit, Department of Rheumatology, Guy’s and St Thomas’ NHS Foundation Trust, London, United Kingdom; 18 Department of Haematology, Barking, Havering and Redbridge University Hospitals NHS Trust, London, United Kingdom; 19 Centre for Rheumatology, Division of Medicine, University College London, London, United Kingdom; 20 Department of Rheumatology, University College London Hospitals NHS Foundation Trust, London, United Kingdom

**Keywords:** antiphospholipid syndrome, ischemic stroke, rivaroxaban, thrombosis, warfarin

## Abstract

**Background:**

Optimal secondary prevention antithrombotic therapy for patients with antiphospholipid syndrome (APS)-associated ischemic stroke, transient ischemic attack, or other ischemic brain injury is undefined. The standard of care, warfarin or other vitamin K antagonists at standard or high intensity (international normalized ratio (INR) target range 2.0-3.0/3.0-4.0, respectively), has well-recognized limitations. Direct oral anticoagulants have several advantages over warfarin, and the potential role of high-dose direct oral anticoagulants vs high-intensity warfarin in this setting merits investigation.

**Objectives:**

The Rivaroxaban for Stroke patients with APS trial (RISAPS) seeks to determine whether high-dose rivaroxaban could represent a safe and effective alternative to high-intensity warfarin in adult patients with APS and previous ischemic stroke, transient ischemic attack, or other ischemic brain manifestations.

**Methods:**

This phase IIb prospective, randomized, controlled, noninferiority, open-label, proof-of-principle trial compares rivaroxaban 15 mg twice daily vs warfarin, target INR range 3.0-4.0. The sample size target is 40 participants. Triple antiphospholipid antibody-positive patients are excluded. The primary efficacy outcome is the rate of change in brain white matter hyperintensity volume on magnetic resonance imaging, a surrogate marker of presumed ischemic damage, between baseline and 24 months follow-up. Secondary outcomes include additional neuroradiological and clinical measures of efficacy and safety. Exploratory outcomes include high-dose rivaroxaban pharmacokinetic modeling.

**Conclusion:**

Should RISAPS demonstrate noninferior efficacy and safety of high-dose rivaroxaban in this APS subgroup, it could justify larger prospective randomized controlled trials.

## 1 Introduction

### 1.1 Background and rationale

Stroke is the second leading cause of death worldwide [1] and the most important cause of adult complex disability [2]. Thrombotic antiphospholipid syndrome (APS) is an acquired autoimmune thrombophilia characterized by venous, arterial, and/or microvascular thrombosis in conjunction with persistent antiphospholipid antibodies (aPL): lupus anticoagulant (LA), immunoglobulin (Ig)G and/or IgM anticardiolipin antibodies (aCL), and IgG and/or IgM anti-beta 2 glycoprotein I (aβ2GP1) antibodies. Among patients with systemic lupus erythematosus (SLE), concomitant thrombotic APS is present in 7% to 15% [3] and confers an adverse prognosis [4,5]. It is estimated that 13.5% (range, 6.8%-23.3%) of individuals with stroke or transient ischemic attack (TIA) have aPL [6]; below the age of 50, approximately 17% (range, 2%-56%) of strokes and 12% (range, 2%-45%) of TIAs are associated with aPL [7]. Ten percent of patients with APS will have a stroke or TIA over 10 years [8]. The spectrum of ischemic brain lesions in APS encompasses white matter hyperintensities (WMHs) of presumed vascular origin and infarcts (subcortical/cortical) [9]. WMHs are an established correlate of small vessel cerebrovascular damage and exhibit face validity, with a systematic review demonstrating their value for predicting increased risk of stroke (hazard ratio [HR]; 95% CI: 3.3; 2.6-4.4), dementia (1.9; 1.3-2.8); and death (2.0; 1.6-2.7) [10]. In patients with aPL, cognitive impairment is common (11%-60.5%) and is associated with WMHs, ischemic lesions, and cortical atrophy [11].

The current standard of care for patients with APS-associated ischemic stroke/TIA or other ischemic damage is anticoagulation with warfarin/other vitamin K antagonist (VKA), with or without an antiplatelet agent. However, owing to a lack of substantive data, optimal antithrombotic therapy in this subgroup of APS patients is uncertain [12]. European Alliance of Associations for Rheumatology (EULAR) guidance [13] gives a range of antithrombotic options for initial ischemic stroke in APS patients: VKA at target international normalized ratio (INR) range of 2.0-3.0 (standard intensity) or 3.0-4.0 (high intensity), taking into account the individual’s risk of bleeding and recurrent thrombosis (level of evidence 1b/grade of recommendation B; Oxford Centre for Evidence-Based Medicine standards). Treatment with standard-intensity VKA plus low-dose aspirin is also included as an option (level of evidence 4/grade of recommendation C).

Warfarin/VKA treatment has several limitations, including recurrent thrombosis, which can occur in APS patients despite therapeutic VKA anticoagulation, with an annualized rate of 4.3% in the prospective Euro-phospholipid cohort of 1000 APS patients [8]. The variable dose-response and narrow therapeutic index of warfarin, along with its numerous drug [14] and dietary interactions, often necessitate frequent anticoagulant monitoring and dose adjustment. In APS patients, INR instability may be exacerbated by increased sensitivity of some thromboplastins to LA and, notably, potential for discordant results with point-of-care INR testing [15]. Direct oral anticoagulants (DOACs) have several advantages over VKA: fixed dosing without need for routine anticoagulant monitoring and considerably fewer drug [16] and dietary interactions. Rivaroxaban [17], a factor (F)Xa inhibitor, along with other DOACs, has emerged as standard of care in general population, with indications including treatment and secondary prevention of a first venous thromboembolism (VTE) [18] and thromboembolism prevention in atrial fibrillation [19].

Current guidance [13,20,21], however, in accordance with recommendations issued by the European Medicines Agency [22] and adopted by regulatory agencies worldwide, cautions against use of DOACs in APS patients with arterial thrombosis and/or triple-aPL positivity (concurrent presence of the 3 criteria aPL: LA, IgG and/or IgM aCL, and aβ2GP1). Two meta-analyses of randomized controlled trials (RCTs) [23,24] in APS patients found a significantly higher risk of subsequent arterial thrombosis during treatment with DOACs compared with warfarin (odds ratios of 5.17 [95% CI, 1.57-17.04] and 5.43 [95% CI, 1.87-15.75] for the other study), although risk of subsequent VTE was not increased. Notably, all previous RCTs of DOACs in APS patients have used standard intensity [[25], [26], [27], [28]] or prophylactic dose [28] DOACs. Further clinical studies are recommended to define the role of DOACs in APS patients, including the use of high-dose DOAC in those with arterial thrombosis [20,21], recognizing the heterogeneity of APS and need for a tailored approach according to thrombotic and laboratory phenotype.

### 1.2 Objectives

This proof-of-principle trial compares the use of high-dose rivaroxaban with high-intensity warfarin for secondary prevention of ischemic stroke or other ischemic brain manifestations in patients with APS. The main objective is to demonstrate noninferior efficacy and lack of major safety signals. Our hypothesis is that high-dose rivaroxaban could represent a safe and effective alternative to high-intensity warfarin in this patient group.

## 2 Methods

### 2.1 Trial design, participants, interventions, and outcomes

The Rivaroxaban for stroke patients with APS (RISAPS) trial is a phase IIb prospective, randomized, controlled, noninferiority, open-label, proof-of-principle trial in adult patients with APS and previous ischemic stroke, TIA, or other ischemic brain manifestations. Eligible patients from participating centers in UK, after providing fully informed written consent, were enrolled and randomized 1:1 to either high-dose rivaroxaban 15 mg twice daily or high-intensity warfarin, target INR 3.5 (range, 3.0-4.0), the latter being regarded as standard of care in this study. The primary outcome for comparison of relative efficacy is the rate of change in brain WMH volume on magnetic resonance imaging (MRI), a surrogate marker of ischemic damage, between baseline and 24 months follow-up between these 2 treatment arms.

The study design was amended from phase II/III to phase IIb following revision of sample size target from 140 to 40 participants (minimum). This was necessitated by adverse recruitment factors, including in relation to the COVID-19 pandemic and decision to exclude triple-aPL-positive patients.

The official title of the trial is: Rivaroxaban vs Warfarin for Stroke Patients With Antiphospholipid Syndrome, With or Without SLE (RISAPS): a Randomised, Controlled, Open-label, Phase IIb, Non-inferiority Proof of Principle Trial.

### 2.2 Participant inclusion criteria

1. Confirmed to have persistent aPL, defined as positivity of one or two aPL, ie, LA, aCL, and/or aβ2GP1 antibodies (IgG and/or IgM) at >40 GPL (IgG phospholipid units) or MPL (IgM phospholipid units) units or >99th centile of normal, on 2 or more occasions at least 12 weeks apart.
2. One or more of a) ischemic stroke; b) TIA with evidence of either acute or chronic ischemic injury on brain MRI—including diffusion-weighted imaging lesion(s), previous cortical or subcortical infarction(s), or WMHs—and diagnosed by a clinician with expertise in stroke; c) brain infarcts (territorial or subcortical) or WMHs of presumed vascular origin on brain MRI, with or without cognitive impairment; and an expert clinical opinion that anticoagulation is a reasonable treatment option (with aim of preventing ischemic brain injury).
3. Body weight ≥ 50 kg and ≤135 kg.
4. Adequate contraception (barrier or hormonal) in women, unless postmenopausal or sterilized.

There was no requirement for the participant’s diagnosis, as per inclusion criteria (1) and (2), to have been made within a certain timeframe prior to enrolment, ie, both *de novo* and established cases were considered. Furthermore, eligibility was not restricted according to the participant’s antithrombotic treatment (active/historical), if any, prerandomization.

### 2.3 Selected participant exclusion criteria

• Age < 18 years
• Triple positivity for aPL (defined as concurrent presence of LA, IgG and/or IgM aCL, and aβ2GP1 antibodies at >40 GPL or MPL units or >99th centile of normal∗)
  • ∗patients previously triple-aPL positive and subsequently single- or double-aPL positive on at least 2 occasions over at least 6 months, including once within 1 month prior to randomization, were not excluded
• Pregnant or lactating women
• Women planning to become pregnant within the 24 month follow-up period
• Severe renal impairment with creatinine clearance < 30 mL/min (Cockroft and Gault)
• Liver function tests: alanine transaminase > 3 × upper limit of normal
• Cirrhotic patients with Child-Pugh B or C
• Thrombocytopenia (platelets <75 × 10^9^/L)

Full exclusion criteria, including contraindicated concomitant medications, are listed in the Supplementary File.

### 2.4 Interventions

Participants were randomized in a 1:1 ratio to either high-dose rivaroxaban 15 mg twice daily (film-coated tablets to be taken orally with food) or high-intensity warfarin, target INR 3.5 (range, 3.0-4.0) for 24 months. Participants on warfarin require INR monitoring by an appropriate anticoagulation clinic in line with usual practice. Adherence to trial medication is assessed by completion of the Medication Adherence Rating Scale (MARS) at each follow-up visit. In addition, for participants on warfarin, INR documentation is reviewed.

The Supplementary File provides further information relating to interventions, including safety processes to mitigate bleeding risk and criteria for anticoagulation review ± modification during trial (including bleeding and thrombosis, weight change, and moderate/severe renal impairment).

### 2.5 Outcomes

The primary outcome is the rate of change in WMH volume (assessed on MRI—a surrogate marker of ischemic damage).

Secondary outcome measures, encompassing neuroradiological and clinical parameters of efficacy and safety, health economics, anticoagulation intensity, and exploratory outcomes including rivaroxaban pharmacokinetic (PK) modeling, are outlined in the Table.

**Table:** Secondary outcomes of trial.

A. Efficacy
1. Neuroradiological markers i) Rates of change in mean diffusivity (assessed on MR imaging) ii) Rates of change in mean fractional anisotropy (as a measure of microstructural white matter damage derived from diffusion tensor imaging) iii) Rates of change on T1-weighted volumetric images of total brain volume iv) Rates of changes on T1-weighted volumetric images of white matter hyperintensities v) Rates of change on T1-weighted volumetric images of grey matter volume vi) Total number of brain infarcts per person as well as the number of these events overall vii) Number of subcortical brain infarcts per person as well as the number of these events overall viii) Number of cortical brain infarcts per person as well as the number of these events overall
2. Clinical i) Vascular events a) Number of ischemic stroke or transient ischemic attacks per person as well as the number of these events overall b) Number of occlusive arterial events at other sites, including systemic embolism per person, as well as the number of these events overall c) Number of cerebral venous thrombosis per person as well as the number of these events overall d) Number of venous thromboemboli at other sites per person as well as the number of these events overall e) Number of microvascular thrombosis per person as well as the number of these events overall f) Number of superficial venous thrombosis per person as well as the number of these events overall ii) Death iii) Composite clinical outcomes a) The number of the composite of all thrombotic events: arterial, venous, microvascular, and death per person, as well as the number of these events overall b) The number of Major Adverse Cardiac and Cerebrovascular events per person, as well as the number of these events overall iv) Rate of change in cognitive function assessed by the Montreal Cognitive Assessment in conjunction with the Queen Square Cognitive Assessment score
B. Safety
1. Bleeding: The number of all bleeding events: major, clinically relevant nonmajor, minor per person, as well as the number of these events overall 2. The Number of serious adverse events other than major bleeding per person as well as the number of these events overall 3. The number of cerebral microbleeds assessed with susceptibility-weighted imaging as a surrogate marker of bleeding risk per person as well as the number of these events overall
C. Health economics
1. Quality of life assessed using 5-level EQ-5D-5L 2. Health and social care resource use assessed using trial follow-up visit case report forms 3. Mean incremental cost per quality-adjusted life year
D. Anticoagulation intensity
1. Rivaroxaban i) Rivaroxaban concentration measured with an amidolytic anti-Xa assay
2. Warfarin i) Time in target INR therapeutic range ii) Factor X level measured with an amidolytic factor X assay (LA independent assessment of warfarin anticoagulant effect)
E. Exploratory outcomes
1. Rivaroxaban pharmacokinetic modeling 2. Cerebral blood flow derived from MR perfusion imaging using an arterial spin labeling technique

INR, international normalized ratio; LA, lupus anticoagulant; MR, magnetic resonance.

RISAPS uses a single scanning site for baseline and outcome MRI brain scans in all trial participants so as to optimize reproducibility and minimize variability in assessment of neuroradiological biomarkers.

To ascertain whether APS patients have a different PK rivaroxaban profile compared with other populations where rivaroxaban is prescribed, we will develop a population PK model for rivaroxaban as part of the RISAPS trial.

### 2.6 Participant timeline

Potential participants were identified at approved hospital trial sites. Written informed consent was required prior to any trial-specific screening procedures. An overview of the trial schedule (including enrolment, interventions, assessments, and visits) is shown in the Figure. Further details of data collection across the trial can be found in Supplementary File.

**Figure:**
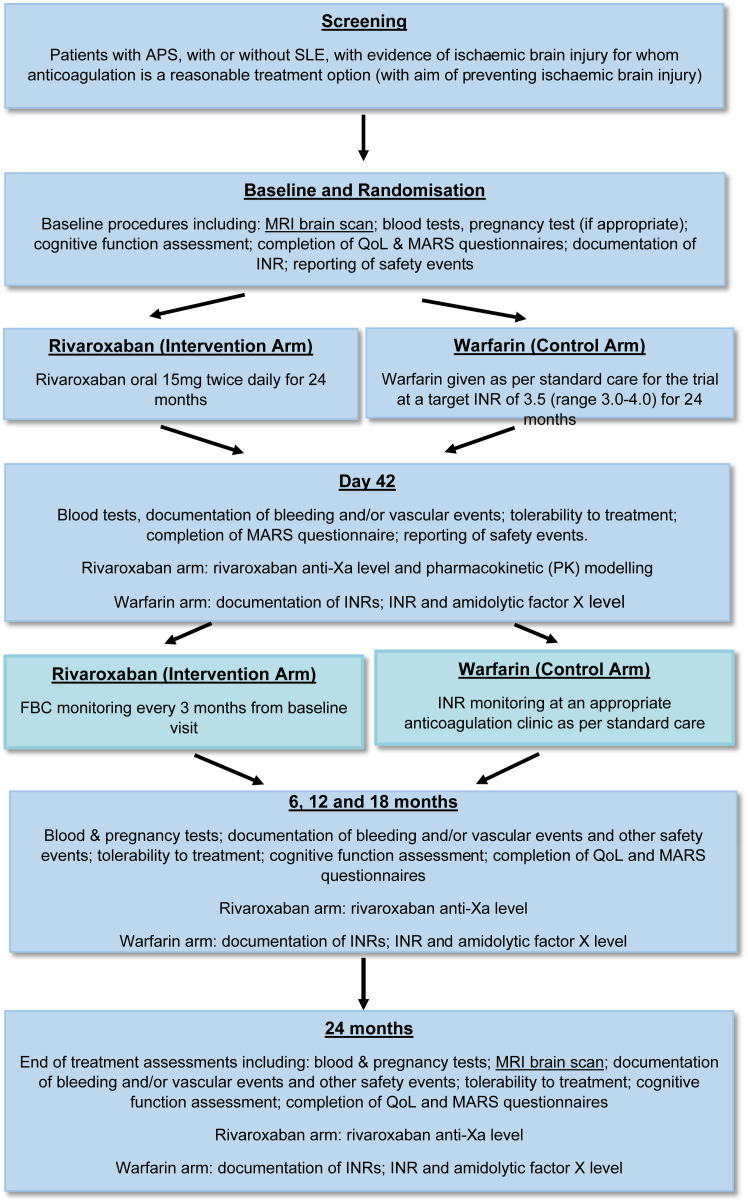
Overview of the trial schedule. APS, antiphospholipid syndrome; FBC, full blood count; INR, international normalized ratio; MARS, Medication Adherence Rating Scale; MRI, magnetic resonance imaging; QoL, quality of life; SLE, systemic lupus erythematosus.

### 2.7 Sample size

The study sample size target was a minimum of 40 participants. RISAPS is a noninferiority trial. Previous research has suggested that noninferiority margin should be half the SD of the primary outcome from historical studies. Accordingly, sample size calculations are based on a study by Benjamin et al. [29]. This used data from the prospective St George’s Cognition and Neuroimaging in Stroke study of patients with symptomatic lacunar stroke and confluent leukoaraiosis (*n* = 121) to determine sensitivity of MRI to change in small vessel disease (SVD) and calculate sample size estimates for a clinical trial. The mean slope (ie, annual rate of change of WMH volume expressed as a percentage of brain volume) was an increase of 0.8% (95% credible interval, 0.67%-0.95%), with a SD of 0.5. To perform a sample size calculation for RISAPS, we have therefore chosen a noninferiority limit of 0.25, ie, the smallest difference in mean slopes between the two groups that would lead us to conclude inferiority. Our noninferiority limit corresponds to half the between-patient variability in slopes. With the planned sample size of 40, we would be able to establish noninferiority based on approximately 50% of this change (a mean increase of 0.4% per year). We have applied a more liberal type I error rate (alpha, falsely declaring a difference when one does not exist) of 10%, which is in line with most phase IIb trials, given that they are not confirmatory in nature.

In practice, 95% CI for WMH volume change will be ±0.32, given the expected SD of 0.5, due to the use of fully observed data as opposed to the formal sample calculation, which adjusted for power of 80%.

### 2.8 Recruitment

Recruitment was monitored monthly, and staff at centers were encouraged and supported to search for suitable patients. Recruitment rates were reported regularly throughout the RISAPS recruiting period to trial oversight committees and funders. Any concerns regarding the rate of recruitment were discussed with relevant committees, and appropriate strategies were implemented.

### 2.9 Assignment of interventions (allocation and blinding)

Participants were randomized by site Principal Investigator/delegate via Sealed Envelope online randomization service, with minimization by SLE status. Due to the requirements of safe anticoagulant management, treatment allocation is not blinded during the study, except at the stage of outcome analysis (brain imaging, neurologic outcomes, bleeding, and recurrent thrombotic events).

### 2.10 Data collection and management

Trial data are collected via a secure online electronic data capture system, and pseudonymized clinical imaging data via a secure file transfer portal.

### 2.11 Statistical methods for analyzing primary and secondary outcomes

The primary outcome will be analyzed using a linear mixed effects model to compare the mean rate of change in WMH volume over time between the two randomized groups. This will allow estimation of average slope in each group with respective 95% CIs, while allowing for variability between individuals within each group. Interparticipant clustering will be modeled by fitting a random intercept model, and measurement of differences in rates of WMH volume changes for each participant will be facilitated by introducing a random slope. The primary model will not include explanatory variables apart from the minimization variable. The fixed effects will include randomized groups only. We will restrict the sample to those who have completed the study and provided data for two MRI scans, as we do not feel that the assumptions of Missing At Random (MAR) or Missing Completely At Random (MCAR) are appropriate in this clinical context.

Further details are provided in the Supplementary File, including (i) methods for additional analyses (eg, subgroup and adjusted analyses) and (ii) definition of analysis population relating to protocol nonadherence and statistical methods to handle missing data.

### 2.12 Monitoring

Central and on-site monitoring is carried out throughout the trial to ensure safety and quality in accordance with requirements detailed in the trial Monitoring and Quality Management Plan. An Independent Data Monitoring Committee monitors the progress of the trial, including patient recruitment, safety events, and interim results.

### 2.13 Study organization and funding

Approvals: The study received clinical trial authorization from Medicines and Healthcare products Regulatory Agency on May 10, 2019, and was approved by the London–Dulwich Research Ethics Committee and Health Research Authority on June 24, 2019 (REC reference 19/LO/0201). Local approvals were also sought from all four participating hospitals.

Dissemination: The results of this trial will be submitted to the funder, as well as for publication in a relevant peer-reviewed journal. Key findings will also be presented at national and international conferences. Published results will be disseminated to investigators at participating sites, who will further disseminate the results to trial participants on request.

The study start date was July 15, 2021 (first patient enrolled), and the planned completion date is February 13, 2025 (last patient last visit). The study is registered at the ISRCTN (International Standard Randomised Controlled Trial Number) registry (reference number 10280992), and ClinicalTrials.gov (ID: NCT03684564).

## 3 Discussion

The RISAPS phase IIb RCT seeks to investigate whether high-dose rivaroxaban can be used safely in APS patients who have had ischemic stroke, TIA, or other ischemic brain injury, with a low rate of ischemic progression on MRI brain imaging over 24 months and absence of safety concerns.

This trial is distinct from previous RCTs of DOAC use in APS patients in that it (i) restricts inclusion to the specific thrombotic subgroup of previous ischemic stroke, TIA, or other ischemic brain injury and (ii) employs a higher-than-standard dose of DOAC, rivaroxaban 15 mg twice daily, intended to be analogous to high-intensity warfarin, target INR 3.5 (range, 3.0-4.0), standard of care in the RISAPS trial.

Previous RCTs have used standard [[25], [26], [27], [28]] or prophylactic [28] intensity DOAC doses and, other than the Rivaroxaban in APS (RAPS) trial [25], all included a heterogeneous population of patients with respect to thrombotic phenotype (venous and/or arterial thrombosis). The RAPS trial restricted inclusion to APS patients with previous VTE requiring standard-intensity VKA. APS patients with arterial thrombosis were excluded, given that there is no precedent (from studies in general population) for the efficacy of standard dose DOACs in the secondary prevention of arterial thrombosis (outside of patients with atrial fibrillation). While DOACs at standard doses have demonstrated comparable efficacy to standard-intensity warfarin in large phase 3 RCTs for VTE [30], with 9% to 10% of such patients diagnosed to have APS [6,31,32], such doses might not be expected to produce an equivalent anticoagulation effect to high-intensity warfarin, a recommended option for APS-associated arterial thrombosis [13]. Rivaroxaban causes dose-dependent inhibition of FXa [33]. PK modeling demonstrates that 15 mg twice daily dose produces higher trough rivaroxaban concentrations compared with standard dose of 20 mg once daily [34], and it might be reasonably expected that the anticoagulation intensity achieved is more comparable with high-intensity warfarin. Notably, animal models indicate that stronger inhibition of FXa by rivaroxaban is required to protect against arterial thrombosis compared with venous [35].

Following EINSTEIN RCTs [36], rivaroxaban is licensed for use at a high-dose of 15 mg twice daily for the first 21 days after a new episode of VTE in the general population (when the risk of recurrence is highest) before reverting to standard maintenance dose of 20 mg once daily. Within these limits, the higher dose of 15 mg twice daily has an established record of clinical efficacy and safety, with a low incidence of major bleeds (0.8%-1.1% in EINSTEIN RCTs, similar to standard-intensity warfarin). Other smaller studies also suggest a potentially favorable safety profile for high-dose rivaroxaban. In a proof-of-concept RCT of rivaroxaban, 15 mg twice daily (*n* = 23) vs dose-adjusted warfarin (*n* = 21) in patients with mechanical heart valves, major bleeding rates were comparably low in each arm after 90 days of treatment [37]. An early phase II study of rivaroxaban in patients with proximal deep vein thrombosis showed no safety signals up to a dose of 30 mg twice daily (major bleeding observed in 1.7%, 1.7%, and 3.3% of patients receiving rivaroxaban 10, 20, or 30 mg twice daily, respectively, across 12 weeks of treatment) [38].

We have chosen a surrogate marker of ischemic brain damage as the primary efficacy outcome measure, namely the rate of change of WMH volume over 24 months, due to the relative infrequency of recurrent clinical thrombotic events in anticoagulated patients with APS. Trials with clinical outcomes and sufficient statistical power have proved challenging in APS patients. The use of surrogate markers in clinical trials has been endorsed in the case of fatal and/or very rare diseases, where validation of hard endpoints may take an unreasonable time to complete [39]. A recent international consensus group reviewed all potential biomarkers for clinical trials in cerebral SVD and concluded that WMH volume remains the most promising established surrogate marker of brain injury in SVD [40].

The follow-up duration of 2 years adopted in RISAPS is based on available data regarding the sensitivity of MRI to the accrual of ischemic brain changes over time. In the prospective St George’s Cognition and Neuroimaging in Stroke study [41], described earlier, changes were detected in multiple MRI markers across 3 years’ follow-up—including WMH volume, but not in cognitive measures. Two years is regarded as an adequate follow-up period to enable meaningful assessment of MRI brain changes, from which a low rate of ischemic progression, alongside favorable secondary clinical outcomes, would support noninferior efficacy of rivaroxaban.

The PK model developed in this trial will be informative for the index population and will also be applicable to other settings where this dose of rivaroxaban might be considered [37].

### 3.1 Summary and conclusions

Appropriately designed studies are required to clarify whether the potential advantages of DOAC as an alternative to warfarin therapy can be extended to certain patient subgroups with thrombotic APS. The RISAPS phase IIb RCT seeks to demonstrate noninferior efficacy and safety of high-dose rivaroxaban vs high-intensity warfarin in patients with APS-associated ischemic stroke, TIA, or other ischemic brain injury, and in so doing, could be a forerunner to larger, potentially practice-changing prospective randomized controlled studies.
