# Supplementary material for: Rivaroxaban for stroke patients with antiphospholipid syndrome (RISAPS): protocol for a randomized controlled, phase IIb proof-of-principle trial
Source: Res Pract Thromb Haemost. 2024 Jun 5;8(5):102468. doi: 10.1016/j.rpth.2024.102468 (PMC11321294; doi:10.1016/j.rpth.2024.102468)
Supplement: Supplementary Appendix [file mmc1.docx]

**SUPPLEMENTARY APPENDIX**

**Contents:**

1. **Full Exclusion criteria**
2. **Trial schedule**
3. **Processes to manage risk of bleeding**
4. **Criteria for anticoagulation review during the trial**
5. **Further information regarding statistical methods**
6. **Changes to the protocol since first approval**
7. **Full Exclusion criteria**

1. Patients who are triple positive for antiphospholipid antibodies (presence of lupus

anticoagulant, anticardiolipin and anti-beta 2 glycoprotein I antibodies (IgG and/or IgM) at >40

GPL or MPL units or > the 99th centile of normal.*

*patients who have previously been triple aPL-positive and have single or double aPL

positivity on at least 2 occasions over at least 6 months, including once within 1 month prior

to randomisation, can be recruited to the trial.

2. Pregnant or lactating women

3. Severe renal impairment with creatinine clearance (Cockcroft & Gault <30

mL/min (i.e. 29 mL/min or less)

4. Liver function tests alanine transaminase (ALT) > 3 x (upper limit of normal) ULN

5. Cirrhotic patients with Child Pugh B or C

6. Thrombocytopenia (platelets < 75 x 10^9^/L)

7. Non-adherence on warfarin (based on clinical assessment)

8. Patients taking strong inhibitors of both CYP3A4 and P-gp pathways such as

a. Systemic azole antifungals (e.g. ketoconazole, itraconazole, voriconazole, posaconazole) b. Patients on human immunodeficiency virus (HIV) protease inhibitors (e.g. ritonavir)

9. Patients on strong CYP3A4 inducers (e.g. rifampicin, phenytoin, carbamazepine,

phenobarbital or St. John's Wort)

10. Patients on dronedarone

11. Patients on levetiracetam, sodium valproate/valproic acid, oxcarbazepine or topiramate

12. Patients less than 18 years of age

13. Refusal to consent to the site informing General Practitioner and Healthcare Professional

responsible for anticoagulation care of the participant

14. Contraindications to MRI (e.g. cardiac pacemaker, severe claustrophobia, inability to lie flat:

patients who do not meet local safety rules for MRI)

15. Patients at high risk of bleeding and not suitable for anticoagulation therapy

16. Previous known allergy or intolerance to warfarin or rivaroxaban

17. Women planning to become pregnant within the 2-year follow-up period

18. Patients with known galactose intolerance, total lactase deficiency or galactose

malabsorption

19. Patients are excluded if they have had active cancer (excluding non-melanoma skin cancers)

within the last 2 years

20. Any other reason that the PI or delegate considers would make the patient unsuitable to

enter RISAPS.

1. **Trial schedule**

|  | **Screening^1^, Baseline & Randomisation** | **Post Allocation** | | | | | |
| --- | --- | --- | --- | --- | --- | --- | --- |
| **TIMEPOINT** | 0 | *Baseline MRI* | *Day 42*  *(+/- 10 days)* | *6m*  *(+/- 14 days)* | *12m*  *(+/- 14 days)* | *18m*  *(+/- 14 days)* | *24m*  *(+/- 14 days)* |
| **ALL PARTICIPANTS** | | | | | | | |
| Consent | X |  |  |  |  |  |  |
| Eligibility screen | X |  |  |  |  |  |  |
| Randomisation | X |  |  |  |  |  |  |
| Medical history and demographics^2^ | X |  |  |  |  |  |  |
| Anti-DNA^3^ | X |  |  |  |  |  |  |
| aPL^4^ | X |  |  |  |  |  |  |
| Height | X |  |  |  |  |  |  |
| Weight | X |  | X | X | X | X | X |
| BMI | X |  |  |  |  |  |  |
| Blood pressure | X |  | X | X | X | X | X |
| Concomitant medication | X |  | X | X | X | X | X |
| Documentation of INRs | X |  |  |  |  |  |  |
| MRI brain scan at Chenies Mews Imaging Centre^5^ |  | X |  |  |  |  | X |
| Baseline blood samples for **local analysis** (FBC, U&E, CrCl, LFTs & INR)^6^ | X |  |  |  |  |  |  |
| Baseline blood sample (14mL) for **centralised analysis**^6^: Citrated plasma & Clotted SST | X |  |  |  |  |  |  |
| Baseline blood samples (26.5mL) for **translational research** (optional): Citrated plasma, EDTA & Clotted SST | X |  |  |  |  |  |  |
| Blood sample (4.5mL) for **centralised analysis^7^**: Citrated plasma |  |  | X | X | X | X | X |
| Blood samples (22.5mL) for **translational research** (optional): Citrated plasma, EDTA & Clotted SST |  |  | X | X | X | X | X |
| Pregnancy test (urine dipstick)^8^ | X |  |  | X | X | X | X |
| Local lipid analysis | X |  |  |  |  |  | X |
| Cognitive function assessments (MoCA & Queen Square measures) | X |  |  | X | X | X | X |
| Quality of life (EQ-5D-5L) | X |  |  | X | X | X | X |
| Medication Adherence Rating Scale (MARs) | X |  | X | X | X | X | X |
| Enquiry for recurrent thrombosis | X |  | X | X | X | X | X |
| Enquiry for bleeding symptoms | X |  | X | X | X | X | X |
| Documentation of AEs^9^ | X |  | X | X | X | X | X |
| Reporting of SAEs^10^ | X |  | X | X | X | X | X |
| Review of tolerability to trial treatment |  |  | X | X | X | X | X |
| Dispensing of Proton Pump Inhibitor | X |  | X | X | X | X | X |
| **RIVAROXABAN PARTICIPANTS ONLY** | | | | | | | |
| PK modelling blood samples (8.5mL; total of 25.5mL if all 3 samples are collected) ^11^ |  |  | X |  |  |  |  |
| Blood samples for **local analysis**: FBC, U&E, CrCl, LFTs^12^ |  |  | X | X | X | X | X |
| Dispensing of rivaroxaban | X |  | X | X | X | X |  |
| **WARFARIN PARTICIPANTS ONLY** | | | | | | | |
| Blood samples for **local analysis**: FBC, U&E, CrCl, LFTs & INR |  |  | X | X | X | X | X |
| Documentation of INRs since enrolment/last visit ^13^ | X |  | X | X | X | X | X |

**Footnotes:**

1. All screening procedures must be carried out within 30 days prior to randomisation
2. Documentation of past thrombosis history; minor, clinically relevant non-major and major bleeding events.
3. Results of anti-DNA should be within 3 months of the screening visit.
4. Results of aPL tests establishing a diagnosis of thrombotic APS, with the dates of when tests were done.
5. Baseline MRI scans to be booked after consent. MRI to be performed as soon as possible after randomisation, in time for participant to start the IMP following randomisation, and within 2 weeks after the baseline MRI scan.
6. Blood results should be within one month prior to consent date. Perform PT/ INR/ your local coagulation test(s) for patients not yet on anticoagulation.
7. Samples will be sent to the UCL Haemostasis Research Unit for centralised analysis. Please see the RISAPS Laboratory Management Plan for more information.
8. Females of childbearing potential only.
9. All AEs should be reported from the time of randomisation until 30 days after the last follow-up (approximately 24 months post randomisation).
10. All SAEs should be reported from the time of consent until 30 days after last follow-up (approximately 24 months post randomisation).
11. PK Modelling venous blood samples at: a) trough (pre-dose) and b) peak (ideally 1 and 3 hours post-dose). Please refer to the Laboratory Management Plan for the type of blood samples required. 8.5mL to be collected per timepoint, and up to a total of 25.5mL if all 3 time points are collected. PK modelling studies are to be done at the Day 42 visit. In exceptional cases, if it is not possible to do the PK modelling studies on Day 42, these studies are permitted to be collected at a subsequent patient visit.

Participants on rivaroxaban should attend for a 3 monthly (+/- 14 days) FBC blood test in addition to the standard trial follow-up visits as part of safety monitoring. In addition to trial follow-up visits, participants on warfarin should continue to attend their routine INR screening.

1. **Processes to manage risk of bleeding**

To manage the risk of bleeding, the following processes are followed for all participants enrolled in RISAPS:

- To minimise the risk of gastrointestinal bleeds, all participants enrolled into the trial are prescribed a proton pump inhibitor (PPI) (lansoprazole 30mg once daily or equivalent).
- All participants on rivaroxaban have three monthly full blood counts (FBCs) to screen for iron deficiency which may be secondary to occult gastrointestinal blood loss.
- All participants are supplied with information, contained within the Patient Information Sheet (PIS) and Participant Adverse Events (AE) diary card, on the symptoms and patient-observable signs of bleeding, and advice on what to do if they experience a bleeding event. All participants who experience bleeding are managed according to local and national/international guidelines.
- Sites are provided with guidance on reviewing of anticoagulation following a clinically relevant or major bleeding episode.

1. **Criteria for anticoagulation review during the trial**

*Bleeding*

Participants who experience a major or clinically relevant non-major bleeding complication during the trial should be assessed by the PI or delegate for subsequent appropriate anticoagulation, based on the ongoing bleeding risk. The UCL CCTU should be informed, followed by discussion between the PI or delegate and CI or delegate, via UCL CCTU, on ongoing management of the patient from the trial treatment perspective.

Participants in the warfarin arm should be considered for the following options: reduction of target anticoagulation intensity such as warfarin target INR 2.5 (range 2.0-3.0), change to subcutaneous LMWH during short-term bleeds with consideration of split dose LMWH, or temporary cessation of anticoagulation.

Participants in the rivaroxaban arm should be considered for the following options: continuation of rivaroxaban 15mg BD if the PI or delegate assess this to be clinically appropriate, change to subcutaneous LMWH during short-term bleeds with consideration of split dose LMWH, or temporary cessation of anticoagulation.

If the participant on warfarin or rivaroxaban experiences a prolonged bleeding episode, including abnormal uterine bleeding, the local PI should review and decide if continued participation in RISAPS is appropriate.

*Suspected thrombosis*

For participants on rivaroxaban who develop recurrent thrombosis, arterial, VTE, or other thrombosis, the rivaroxaban will be stopped, and the patient will be commenced initially on subcutaneous LMWH, generally at standard therapeutic dose (or fondaparinux in participants known to have allergy or intolerance to LMWH), with expert clinical review at the participating centre. A similar approach will be undertaken in patients on warfarin. Further anticoagulant management will be decided by the clinician responsible for the participant.

*Changes in weight*

If a participant experiences a reduction in weight of 10% or more between two trial follow-ups, the UCL CCTU should be informed and the PI or delegate should ensure appropriate clinical review (e.g. by the GP) and, if on rivaroxaban, also closer monitoring of weight between trial visits. If a participant on rivaroxaban experiences a weight reduction to less than 50kg, or a weight increase to over 135kg, further management of the participant from the trial treatment perspective should be discussed between the PI or delegate and CI or delegate, via the UCL CCTU.

*Moderate or severe renal impairment*

Participants in the rivaroxaban arm of the trial who develop moderate renal impairment (Creatinine clearance 30 – 49 mL/min) during the trial, after randomisation, will require discussion, between the PI or delegate and CI or delegate, via the UCL CCTU, on management of the patient from the trial treatment perspective. In the event that a participant develops severe renal impairment while on the trial, (creatinine clearance <30 mL/min [i.e. 29 mL/min or less]) they should be withdrawn from trial treatment but continue trial follow up.

1. **Further information regarding statistical methods**

*Methods for additional analyses (e.g subgroup and adjusted analyses)*

While the factor “SLE status at baseline” was used as a minimisation factor in the randomisation algorithm, we do not anticipate that we will have recruited sufficient numbers of eligible participants to provide sufficient power to warrant any subgroup analyses.

We will report per protocol analyses and the results of all supportive sub analyses, sensitivity analyses and threshold analyses as appropriate.

Further post-hoc subgroup analyses looking at the relative effects of the intervention may be carried out, if appropriate, for the following subgroups:

Age

Sex

Type of initial stroke

BMI

Smoking

Presence/absence of hyperlipidaemia

Presence/absence of diabetes mellitus

Presence/Absence of hypertension

Single of double antiphospholipid antibody positivity

We will perform accompanying secondary analyses in which we introduce data from participants who have missing data and models which include the covariates of age, sex and SLE status inter alia to assess the stability of the results to different modelling environments. We will assess the results with respect to comparable analyses of covariance (ANCOVA) models. Where appropriate, we will report the results of the comparative supportive analyses (either in the primary report of the outcome of the study, or in separate journal articles which focus on the innovative methodological techniques employed in the analyses of these data.

*Definition of analysis population relating to protocol non-adherence and statistical methods to handle missing data*

We will use a completers-only analysis for the primary outcome (i.e., the analysis population will be limited to those participants who provided analysable data from MRI scans at both timepoints required in the treatment protocol). Supportive analyses will employ all available data and use multiple imputation by chained equations where possible.

1. **Changes to the protocol since first approval**

| Version amended | Reason for Amendment |
| --- | --- |
| Version 1.0 | - At the request of the MHRA some administrative changes were made, to commence reporting of Adverse Events from the time of consent and change of wording from 1 day to 24 hours regarding timelines for events reporting. - Expectedness of SAE assessed by the sponsor clinical reviewer only - Update to the wording for the health economics analysis and administrative change to the Health Economic section to allow clearer delineation from the main trial analysis. - Administration changes to the NAE reporting section to make timelines for sites reporting events clearer. - Appendix 4 removed, readers are referred to the current and approved versions of SPCs for the study (section 4.8) |
| Version 2.0 | - Participant timeline amended to make trial assessments clearer and to distinguish between treatment arms. - Information on EMA recommendation regarding the use of DOACs added. - Advice from the new EULAR guidelines regarding the optimal INR range based on the risk of bleeding and recurrent thrombosis has been incorporated. - Minor change to wording in inclusion criteria. - Removed requirement to include rivaroxaban manufacturer’s patient information leaflet as per the advice of the Sponsor. - Regular safety reports, initially monthly, to the Independent Data Monitoring Committee (IDMC) added to address the need for additional safety measures due to the EMA recommendation. - The requirement to collect pregnancy outcomes, should a participant fall pregnant while on the trial or in follow up, has been incorporated in the protocol. - Clarification on NAE reporting - St George’s Hospital has been added as a new participating site with Dr Arvind Kaul as Principal Investigator - Lists of members of the IDMC and TMG have been updated. - Administrative changes throughout the protocol |
| Version 3.0 | - Date of first enrolment amended to March 2020 to reflect amended timelines - Exclusion criterion 17 added which clarifies that participants should be excluded if the PI assesses that there is any other reason, not already listed in the exclusion criteria, which would make the participant unsuitable for trial treatment. - All references to enrolling participants who require standard-intensity anticoagulation (standard dose rivaroxaban (20mg daily) or standard intensity warfarin (target INR 2.5, range 2.0-3.0) at enrolment, if they are deemed unsuitable for higher intensity/ dose treatment, has been removed. - The following statement has been added in relation to prescribing ranitidine, for patients who are allergic to or intolerant of PPIs: Participants allergic to or intolerant to PPIs, will be prescribed ranitidine (if available) or a suitable equivalent, in accordance with local policy at the trial site. - Section 6.4.4.1 (Criteria for anticoagulation review during the trial) has been reworded to remove the option of dose reduction of rivaroxaban to 20mg once daily in patients who experience major or clinically relevant, non-major bleeding on high-dose rivaroxaban (15mg twice daily) and are assessed as suitable for standard-intensity anticoagulation. The following alternative options have also been provided for patients who experience bleeding: subcutaneous LMWH during short-term bleeds with consideration of split-dose LMWH, or temporary cessation of anticoagulation. *(nb. This change was made prior to active enrolment of participants).* - The existing table which outlines initiation/ switching regimens at enrolment has been amended and new information added for participants on subcutaneous LMWH randomised to warfarin, participants on an antiplatelet agent randomised to rivaroxaban or warfarin and for patients not on anticoagulation or an antiplatelet agent, randomised to rivaroxaban or warfarin. - Participants are advised to attend their nearest A&E/ emergency department immediately in the event of a suspected thrombosis or bleed (or other emergency). Previously the advice given was to attend their nearest A&E department. - It has been clarified that in case of weight reduction of 10% or more between trial visits, or a weight reduction to less than 50kg, the UCL Comprehensive Clinical Trials Unit (CCTU) should be informed to discuss the clinical management of the patient. - Clarification has been added that CCTU will provide information relating to the pregnancy for participants on rivaroxaban only. - Reference to recirculating unused rivaroxaban in to general hospital stock has been removed as there is no arrangement for this. - Antiepileptic medications have been added to medications to be used with care and to pre-randomisation notifications to UCL CCTU - Clarification on dispensing has been added - Section 6.9.2.1 has been updated with additional information in relation to the handling of imaging data. - This is the fourth version of the RISAPS protocol and there have been three substantial amendments to the original protocol. - Minor administrative changes have been made throughout the document. |
| Version 4.0 | - Administrative changes to TMT,TMG, TSC and IDMC - Clarification added to 5.1 Background & rationale - Clarification added to 6.3.1.7 Screening Procedures and Pre-randomisation Investigations regarding enrolment of triple aPL-positive |
| Version 4.1 | - Provided further information on assessing triple positivity aPL diagnosis - Added to the exclusion criteria:   - triple positive aPL antibodies   - weight restriction   - sodium valproate/valproic acid and topiramate   - patients who have had active cancer - Site to discuss and/or notify UCL CCTU on the following:   - Pre-existing moderate renal impairment or development of moderate or severe renal impairment during the trial   - Participants taking antiplatelets, long-term NSAIDs, SSRI or SNRI during the trial   - Severe or persistent bleeding or symptoms of thrombosis   - Significant changes in weight - FBC (for patients on rivaroxaban) can be collected outside of recruiting site if needed - Updated guidance in initiating/switching regimens - Clarified participant guidance if they experience severe or persistent bleeding or symptoms of thrombosis to see their A&E hospital. Guidance is also given to site on how to manage their trial medication regimen. - Added new trial results published recently to the trial background - Clarified that safety reports sent to IDMC will only continue unless otherwise indicated by the IDMC - PK modelling blood samples can be collected at next visit if missed at Day 42 - Moved the ancillary and post-care section further up the protocol - Clarified what information will be documented in the MRI research governance report - Updated the EDC section that sites can remotely enter data into MACRO - Added if the participant contracted COVID-19 and meets the SAE definition, expedited reporting is required - Added a ‘delegate’ for CI or PI - Updated the reference list - Administrative changes to TMT, TMG, and IDMC - Minor administrative changes have been made throughout the document |
| Version 5.0 | - Trial changed to Phase IIb, proof of principle trial and wording relating to this changed throughout protocol. - Signatories for protocol updated - Sample size amended from 140 patients to 40 patients, wording changed throughout protocol. - Sections 1.3 and 5.2.1 Clarifications added around Primary, secondary and exploratory outcomes - Roles and responsibilities in Section 1.4 updated to add / remove trial staff. - Section 5.1.1 updated to state that DOACS have fewer drug-food interactions and significantly fewer drug-drug interactions. Previously stated no drug interactions. - Section 6.4.3 Dispensing: Added in sentence permitting use of hospital stock of rivaroxaban in exceptional circumstances with prior approval from the sponsor and Chief investigator. - Section 6.4.9 (2) stopping guidelines for safety updated to include lower expected number of thrombotic and bleed events based on a reduced sample size of 40 patients. - Section 6.5 PK modelling blood samples can be collected at subsequent visit if missed at Day 42. Previously stated ‘next visit’ after Day 42. - Section 6.6 Sample Size: updated to reflect statistical reasoning for reduced sample size of 40 patients. - Section 6.8.1.1 Sequence generation: Sentence added around minimisation used in the study. - Section 6.9.2.1 Section updated with reference to the transfer and format of the MRI data to Kings College for analysis. - The statistical analysis of the trial is updated in Sections 6.9.4.2, 6.9.4.4 and 6.9.5 to account for the change to a phase IIb and a reduction in sample size from 140 to 40 participants. - Section 6.9.4.3 Additional Analyses -Subgroup updated to specify the different sub-group analyses that may be carried out including; Age, Sex; Ethnicity, Qualifying inclusion criteria, cerebral microbleeds at baseline, concurrent antiplatelet use, atrial fibrillation, BMI, Smoking, presence/absence of hyperlipidaemia, diabetes, hypertension, single or double antiphospholipid antibody positivity. - Section 6.9.6 health economic evaluation updated based on reduction in trial sample size. - Section 6.10.2: removal of IDMC review of sample size calculation assumptions as no longer applicable due to reduction in sample size. - Updated the reference list - Minor administrative changes have been made throughout the document. |
